# The Roles of Skin Langerhans Cells in Immune Tolerance and Cancer Immunity

**DOI:** 10.3390/vaccines10091380

**Published:** 2022-08-24

**Authors:** Li Zhou, Aimin Jiang, Jesse Veenstra, David M. Ozog, Qing-Sheng Mi

**Affiliations:** 1 Center for Cutaneous Biology and Immunology Research, Department of Dermatology, Henry Ford Health System, Detroit, MI 48202, USA; 2 Immunology Research Program, Henry Ford Cancer Institute, Henry Ford Health, Detroit, MI 48202, USA; 3 Department of Biochemistry, Microbiology, and Immunology, School of Medicine, Wayne State University, Detroit, MI 48202, USA; 4 Department of Internal Medicine, Henry Ford Health, Detroit, MI 48202, USA

**Keywords:** Langerhans cells (LC), dendritic cells (DC), tissue-resident macrophages (TRM), immune tolerance, cancer immunity, nonmelanoma skin cancers

## Abstract

Langerhans cells (LC) are a unique population of tissue-resident macrophages with dendritic cell (DC) functionality that form a network of cells across the epidermis of the skin. Their location at the skin barrier suggests an important role for LC as immune sentinels at the skin surface. The classification of LC as DC over the past few decades has driven the scientific community to extensively study how LC function as DC-like cells that prime T cell immunity. However, LC are a unique type of tissue-resident macrophages, and recent evidence also supports an immunoregulatory role of LC at steady state and during specific inflammatory conditions, highlighting the impact of cutaneous environment in shaping LC functionality. In this mini review, we discuss the recent literature on the immune tolerance function of LC in homeostasis and disease conditions, including malignant transformation and progression; as well as LC functional plasticity for adaption to microenvironmental cues and the potential connection between LC population heterogeneity and functional diversity. Future investigation into the molecular mechanisms that LC use to integrate different microenvironment cues and adapt immunological responses for controlling LC functional plasticity is needed for future breakthroughs in tumor immunology, vaccine development, and treatments for inflammatory skin diseases.

## 1. Introduction

Langerhans cells (LC) are a unique population of mononuclear phagocytes in the skin epidermis that arise from embryonic macrophage precursors [1] and are highly conserved across vertebrate species [2]. Although LC were originally considered a subset of dendritic cells (DC) due to their capability of migrating to the skin-draining lymph nodes, recent lineage tracing, and ontogeny studies have demonstrated that LC are instead a subset of tissue-resident macrophages (TRM) that acquire a DC-like phenotype and function upon further differentiation in the skin [3,4]. Localization in the skin, which is the outermost interface between the body and the environment, indicates the strategic importance of LC as immune sentinels and a first line of immunological defense. Over the past few decades, the scientific community has extensively studied the DC-like immunogenicity of LC. However, as a type of macrophages, a more prominent immunoregulatory role of LC at steady state and within certain inflammatory conditions is revealed in recent studies. In this mini review, we summarize the recent literature on the immune tolerance function of LC during homeostatic and disease conditions, including skin cancer development and progression, and we focus on results obtained from murine system. Additionally, the microenvironmental cues promoting LC functional plasticity and the potential connection between LC population heterogeneity and functional diversity will likewise be discussed.

## 2. Langerhans Cells in Ultraviolet Radiation-Induced Immune Suppression

Exposing cells and animals to haptens and ultraviolet (UV) light has long been the most popular strategy for studying LC migration and activation. UV radiation, particularly UVB, is the major risk factor for skin cancer, and it induces skin inflammation and immunosuppression.

Exposure to UV light results in extensive DNA damage in keratinocytes (KC), leading to multiple possible fates including enzymatic DNA repair; apoptosis-mediated elimination; metabolic stress-induced NKG2D ligand surface expression, followed by natural killer (NK) and T cell mediated elimination; and the acquisition of mutations within cell cycle regulatory and stimulatory genes that endow apoptosis resistance and clonal persistence [5,6,7].

The migration of LC into lymph nodes and the induction of antigen-specific CD4^+^CD25^+^Foxp3^+^ regulatory T cells (Treg) following UV exposure has been shown to be an important mechanism for UV-induced immunosuppression [8]. In contrast, one study has shown that dermal langerin^+^ cells, but not LC, are responsible for the UV-mediated suppression of later stage CD8^+^ T cell expansion in response to both contact hypersensitivity and epicutaneous protein immunization [9]. These dissimilar findings may be related to animal model and methodological specificities, as well as the phase of disease under study [10]. As a special type of TRM with DC functionality, the involvement of LC has been implicated as being involved in a number of Treg-dependent skin immune suppression models. However, clarifying the mechanisms and locations of the cellular interactions between LC and Treg is still needed.

After exposure to UV radiation, the LC-mediated phagocytosis of apoptotic KC is essential for the anti-inflammatory role of LC in the resolving of UV-induced cutaneous inflammation [11], which can be initiated by the autocrine/paracrine anti-inflammatory factors, such as transforming growth factor beta (TGF-β), prostaglandin E2 (PGE2), and platelet activating factor (PAF), induced by apoptotic cell ingestion [12,13,14]. The interaction of the receptor activator of nuclear factor κ B (RANK) with RANK ligand (RANKL) between LC and inflamed KC has been shown to play a critical role in UV-induced immunosuppression, which results in an increased capacity of LC to induce an IL-10-driven CD4^+^CD25^+^ Treg response [15]. This result suggests a potential role for LC in directly regulating effector T cell immunity in local skin tissue. This concept is supported by another study which showed that apoptotic keratinocytes upregulate RANKL, which induce IL-10 production by LC after UVB radiation [16]. The same study also showed that the expression of OX40L by LC and the interaction between LC and T cells by OX40L-OX40 is essential for antigen-specific Treg responses [16]. However, in contrast to the RANK–RANKL interaction between LC and apoptotic KC that results in IL-10 production and Treg accumulation, a more recent study indicates that the interaction between CD300a^+^ LC and apoptotic KC or members of the gut microbiome restricts the numbers of local Treg cells, which is required for the control of *S. typhimurium* in the gut but could cause deleterious inflammatory responses in the skin [17]. These results indicate the context-dependent immune regulatory role of LC and a balanced regulatory role for LC in epithelial tissue immune homeostasis.

In addition to the LC-mediated Treg expansion and the related immune suppression effects, Lewis et al. showed that LC-intact epidermis develops UVB-induced tumors more readily than LC-deficient epidermis and LC facilitates the clonal expansion of p53 mutant KC, in a process that is completely αβ and γδT cell-independent [18]. These data demonstrate that LC play a key role in UVB-induced cutaneous carcinogenesis, suggesting that LC locally stimulate KC proliferation and that innate immune cells may mediate the tumor outgrowth. Furthermore, a recent study has indicated that LC facilitate a shift in epidermal RORγt^+^ IL-22^+^ IL-17^+^ innate lymphoid cells (ILC) in association with UV-Induced p53 Mutant KC Expansion [19], an additional mechanism of LC-mediated immune regulation that facilitate UV induced tumorigenesis. Studies on the role of LC in UV-mediated immune suppression are summarized in Table 1.

## 3. Langerhans Cells in Steady State Skin Immune Homeostasis

A key role of TRM is to maintain immune homeostasis within tissues, and this task is exercised partly through their scavenger function of rapidly clearing debris from dying cells under both steady state and pathogenic conditions. Like other TRM, LC employ a similar innate mechanism to control local immune tolerance in the skin [20]. Phagocytosis of apoptotic cells by macrophages and DC leads to inflammatory immune response suppression [12,21]. Transforming growth factor-β1 (TGF-β1) is a fundamental regulator of immune cell development and function and this molecule is a crucial regulatory factor for LC development, maintenance, and function with context-dependent down-stream signaling pathways [22,23,24]. Axl is a TAM (Tyro3, Axl, and Mer) receptor tyrosine kinase family member, which functions as an inhibitor of innate inflammatory responses in dendritic cells and is essential for the prevention of lupus-like autoimmunity [25]. TGF-β1-induced LC differentiation is accompanied by Axl upregulation, which enhances the phagocytosis of the Axl ligand (Growth arrest specific 6, GAS6)-expressing apoptotic KC and the inhibition of inflammatory cytokine production [26]. Meanwhile, TGF-β1-independent inflammation induces Axl upregulation, which could provide a negative feedback control of proinflammatory cytokine production [26,27,28]. Although adult human skin contains 10 to 20 billion resident memory T cells, resting LC selectively induces the activation and proliferation of skin-resident Treg, whereas in the presence of foreign pathogen, the activated LC induce the proliferation of effector memory T cells but limit Treg activation [29].

**Figure 1:**
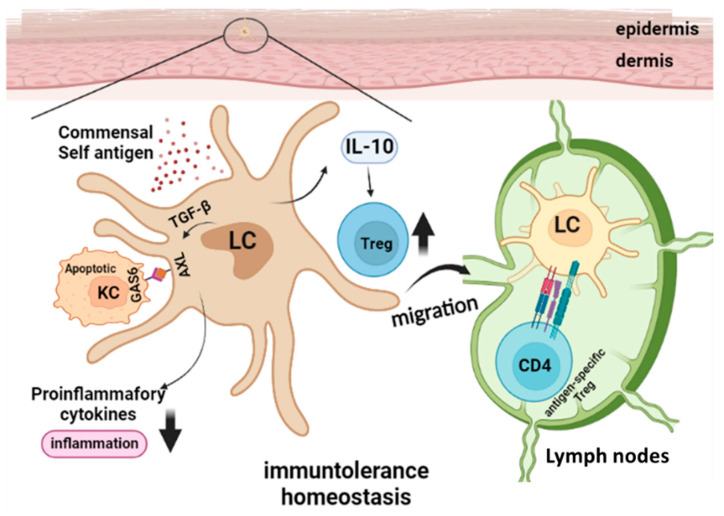
**Langerhans Cells in Steady State Skin Immune Homeostasis.** In homeostatic conditions, LC constantly migrate to draining Lymph nodes and present self or commensal antigens and drive the development of Treg, while skin local Treg amplification can also be induced by IL-10 produced by LC. Transforming growth factor-β1 (TGF-β1)-induced LC differentiation is accompanied by Axl upregulation, which enhances the phagocytosis of Axl ligand GAS6-expressing apoptotic KC and the inhibition of inflammatory cytokine production.

These results indicate a specific role for LC in maintaining immune tolerance in normal skin, as well as for activating a protective immune response upon infectious challenge. Notably, non-activated LC constantly migrate to draining lymph nodes to present self-antigen and establish immune tolerance during homeostatic conditions [30]. As frontline immune sentinels in the skin, LC reside in close contact with the bacterial skin flora. It is attractive to hypothesize an immune tolerogenic role for LC to prevent reactivation of bacteria-specific memory T cells that are present in the skin. LC have been shown to have a relatively low capacity to internalize and process bacteria and skin commensal bacteria-primed LC drive the development of Foxp3+ Treg [31]. Furthermore, the long-term absence of epidermal resident LC leads to significant gene expression changes in the local KC and resident dendritic epidermal T cells, indicating that LC have an active role in maintaining epidermal tissue homeostasis [32]. The roles of LC in steady-state skin homeostasis are shown in Figure 1.

## 4. Langerhans Cells in Skin Treg Differentiation and Inflammatory Disease Models

TRM play an important role in the suppression of local adaptive immunity by direct and indirect interaction with Tregs. TRM from multiple tissues, such as small intestine [33,34], lung [35,36], liver [37,38,39], and brain [40], induce the differentiation of Treg through the production of Treg-promoting cytokines, including IL-10, TGF-β, and retinoid acid (RA). In addition, antigen-presenting DC have the capacity to promote Treg differentiation in a similar fashion via the secretion of mediators, such as TGF-β and RA [41,42,43]. Steady-state LC encountering self-antigens induce Treg differentiation [44], whereas foreign antigens will promote an inflammatory cascade. In addition to facilitating cytokine mediated Treg differentiation and functional promotion, TRMs interact directly with Treg in situ. A model of experimental autoimmune encephalomyelitis showed that sialoadhesin (Sn)^+^ TRM directly interact with Treg through a dialogue between Sn^+^ macrophages and Sn-accessible sialic acid residues on Treg, which impact local immune suppression and disease progression [45]. These results suggest that inducing Treg differentiation is a common strategy shared by TRM and DC, including LC, in maintaining immune tolerance and local tissue homeostasis.

As a special type of TRM, LC have been directly implicated in a number of Treg-dependent models of immune suppression in the skin. Contact hypersensitivity (CHS) is an experimental model of allergic contact dermatitis that can be studied in mice [46]. To study the role of LC in the pathogenesis of allergic contact dermatitis, a variety of LC depletion mice have been used as models of CHS [47,48,49,50,51,52,53,54,55]. Indeed, LC were shown to be essential for establishing tolerance to mild contact allergen sensitization by activating and expanding Treg and inducing allergen specific CD8+ T cell tolerance [51,53,54,55]. In support of this finding, the transgenic over-expression of RANKL by KC inhibits CHS responses by augmenting the capacity of LC to expand Treg [15]. In a grafted skin model of CHS, IL-10-producing LCs that had been stimulated by RANKL produced by the grafted skin were shown to induce Treg cells, which helped quell the cutaneous inflammation [56]. Furthermore, transgenic mice expressing CD1a, a lipid-presenting molecule that normally is not present in mice, showed the suppression of CHS in response to dinitrofluorobenzene, whereas these CD1a+ LC were able to induce Th17 responses to topical inflammatory plant lipids [57]. This result indicates that the molecular nature of the haptens may play a role in determining the direction of LC-mediated immune responses, and this idea is supported by the fact that resting LC selectively induces the activation and proliferation of skin resident Treg, whereas in the presence of foreign pathogens, activated LC induce the proliferation of effector memory T cells and limit Treg activation [29]. However, these conclusions have been challenged by other reports that have shown mitigated CHS responses in LC deletion mouse models [47,52]. Multiple possible explanations to these seemingly conflicting results are possible, since LC and dermal DC have functional redundancy in CHS [49,50]. For instance, the murine Langerin promoter-mediated gene mutations occur in both LC and Langerin-expressing dermal DC [58], while human Langerin promoter-mediated gene mutations are LC-specific [51], which may be one reason why diversified outcomes in the CHS models have been seen. Additionally, the experimental dose, route, and location of hapten application could also affect how deeply the haptens penetrate the skin (epidermis vs. dermis) and could result in different types of DC involvement in the observed CHS responses. In patients with atopic dermatitis, the situation could be much more complex. At the very least, experimental conditions, such as the nature, dose, and route of allergens, as well as the skin inflammatory conditions, would play a role in determining the nature of the LC response. In addition to IL10 production, human LC exhibit regulatory function through the aryl hydrocarbon receptor (Ahr) [59] and resting LC from human skin selectively induce the activation and proliferation of skin resident Tregs in vitro [29]. These results could explain the finding of Igyarto et al. [55] where LC-deficient mice were more prone to contact hypersensitivity reactions than control mice.

To explore the role of LC in immuno-regulatory responses within a physiological setting, Kitashima et al. used the KC-associated autoantigen Desmoglein 3 (Dsg3)-specific experimental autoimmune dermatitis (EAD) model. They found that LC take up Desmoglein-3 via langerin and present it through MHC-II molecules to induce Treg proliferation, in which IL-2 produced by non-Treg cells is required for both LC functional condition and subsequent Treg cell expansion [60]. This finding suggests an IL-2 signaling-dependent LC-mediated Treg expansion leads to peripheral tolerance against an epidermal autoantigen.

The skin hosts a variety of DC to form a complex skin DC network. Using a LC-specific inducible neoantigen expression mouse model, a recent study evaluated LC-specific contributions to immune activation and tolerance pathways. In this model, the presentation of endogenous ovalbumin (OVA) in steady state LC led to the priming of CD8^+^ T cells in lymph nodes (LN) and the accumulation of cutaneous CD4^+^ Treg and Ag-specific cytotoxic T lymphocyte (CTL) tolerance in the recall reaction. By contrast, when OVA was presented by activated LC in transgenic mice, a recallable CTL memory response developed [61]. Thus, neoantigen presentation by epidermal LC results in either robust CTL tolerance or CTL memory, and this decision-making depends on the activation state of the presenting LC [61], revealing the essential involvement of local environment in the functional flexibility of LC. The requirement of antigen uptake by LC for the induction of regulatory T cells and the acquisition of tolerance during epicutaneous immunotherapy in OVA-sensitized mice further indicate the essential role of LC in skin immune tolerance within the skin DC network [62,63].

In LC generated from CD34^+^ hematopoietic stem cells, aryl hydrocarbon receptor (AhR) activation by FICZ, a potent AhR agonist, reduces FcεRI and upregulates Indoleamine 2,3-dioxygenase (IDO) expression. This AhR-mediated anti-inflammatory feedback mechanism may dampen allergen-induced inflammation in atopic dermatitis (AD) [59]. A recent study showed the defective TLR2-mediated sensing of *S. aureus*-derived signals by LC and inflammatory dendritic epidermal cells in AD patients, which may partly contribute to the immune deviation and the lack of *S. aureus* clearance in these patients [64].

The role of LC in psoriasis is currently debatable since previous reports have shown decreased [65,66], increased [67,68], and even unchanged [69,70] quantities of LC in psoriatic lesions from patients. The proinflammatory role of LC in psoriasis pathogenesis has been shown in numerous studies within different psoriasis models, especially the imiquimod-induced (IMQ) psorasis-like dermatitis model. These studies suggest a potential positive feedback loop between keratinocytes, LCs, and T cells during the development of psoriasis [70,71,72,73,74]. In this process, an altered KC secretome, driven by the upregulated expression of IL-17 in the skin milieu, is responsible for impaired LC migration [75]. STAT3 activation in KC promotes LC IL-23 production via the p38α signaling pathway [71,72], and the IL-23 produced by LC activates skin γδT and other T cells to express and release IL-17, which in turn changes or enhances the KC secretome, ultimately resulting in the activation and retention of LC.

**Figure 2:**
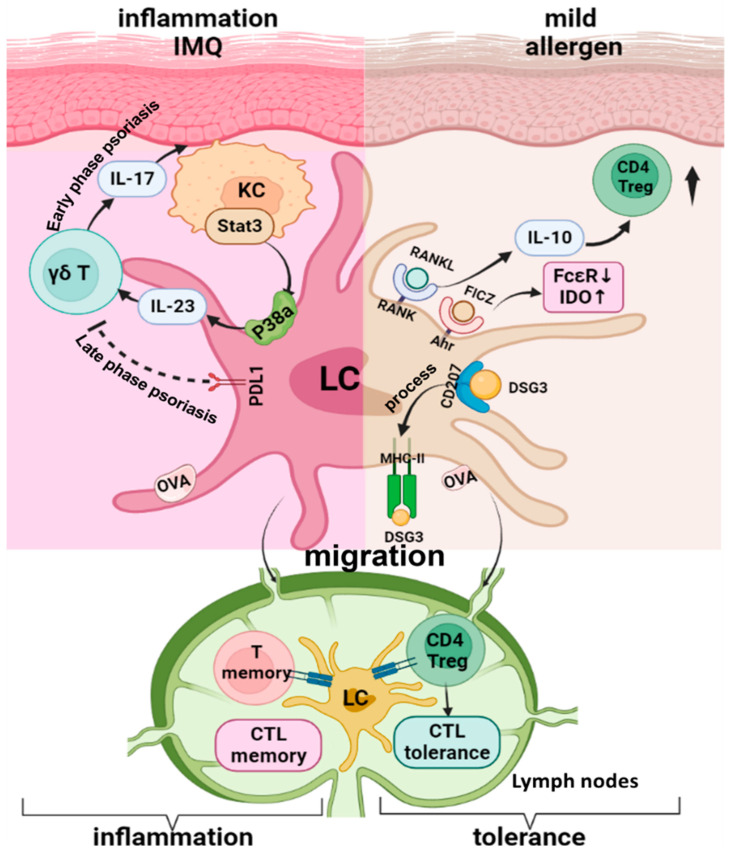
**Langerhans Cells in Inflammatory Skin Diseases**. **Right panel** of the figure showed the tolerance induction function of LC in mild allergen induced contact hypersensitivity (CHS). RANKL expressed by KC inhibits CHS responses by augmenting the capacity of LC to expand Treg. Aryl hydrocarbon receptor (AhR) on human LC can be activated by agonist FICZ, which reduces FcεRI, upregulates Indoleamine 2,3-dioxygenase (IDO) and reduced inflammation. LC take up Desmoglein-3 (DSG3), a KC associated autoantigen, via Langerin (CD207) and present it through MHC-II to induce Treg proliferation. **The left panel** showed the pro-inflammatory role of activated LC under inflammatory milieu. In IMQ induced psoriasis dermatitis model, STAT3 activation in KC promotes LC IL-23 production via the p38α signaling pathway, the IL-23 produced by LC then activate skin gdT and other T cells to express and release IL-17 which in turn enhance the KC inflammation. However, in the late phase the IMQ model, LC-specific PDL-1 can help to ease the skin inflammation via PD1/PDL-1 axis. In a LC-specific inducible ovalbumin (OVA) expression mouse model, presentation of endogenous OVA by activated LC induces a recallable cytotoxic T lymphocyte (CTL) memory response, whereas OVA presentation by steady state LC results in Treg accumulation and CTL tolerance.

To more closely recapitulate the clinical and cellular hallmarks observed in patients with psoriasis, Malissen and Henri’s group used a 14-day consecutive IMQ-induced psoriasis-like dermatitis mouse model to evaluate the dynamic changes of skin DC and macrophages during both the early-onset and the late-stable phases of psoriasis. Their results indicate that LC depletion leads to an increased number of neutrophils in the late phase of inflammation, suggesting a regulatory role of LC in the late stage of IMQ-induced skin inflammation [76]. This result is supported by a more recent study, which showed that mice with LC-specific PDL1 deletion presented significantly more severe IMQ-induced dermatitis and increased IL-17 producing γδT cell activity in the skin [77]. These results indicate a functional flexibility in LC for the inflammation initiation versus the maintenance of IMQ-induced psoriasis-like dermatitis, in which the PD-1/PD-L1 axis is involved in the immune regulatory function of LC in the late stage of inflammation. Detailed molecular mechanisms that enable the functional shifting of LC during the process of inflammation remain to be elucidated. Figure 2 is the schematic showing the plasticity of LC function in inflammatory skin diseases.

## 5. Langerhans Cells in Skin Cancer Development and Progression

In the mouse epidermis, LC and dendritic epidermal T cells (DETC) form spatially complementary DC networks. Dendrites from the LC-DETC network extend to contact each other and most basal KC [30,78], which is where epidermal malignant transformation typically occurs. Furthermore, LC constantly survey and respond to epidermal stress and trigger downstream inflammatory and immunogenic responses. Thus, mouse skin serves as a model for studying epithelial/immune cell interactions and their relevance in cancer [79], since all epithelial tissues are variably populated by local DC and resident T cells. The critical role of cutaneous immune surveillance for preventing of skin cancer development is best demonstrated among patients with solid organ transplants, who have a substantially increased risk of developing skin cancer, especially squamous cell carcinoma, because of the prolonged immunosuppressive regimen required to prevent organ rejection [80]. This concept is further supported by the presence of a comparable burden of somatic driver mutations occurring in skin cancer cells and adjacent normal skin, indicating that while malignant conversion is occurring at both sites, it remains in check fromimmune surveillance in the normal skin region [81,82].

LC have traditionally been considered antigen-presenting cells (APCs) that activate adaptive immune responses. Recent studies have confirmed the capacity of LC to process and present antigens to activate the CD4 helper T cell and CD8 cytotoxic T cell compartments, including potent antitumor effector cells. The epicutaneous accessibility and immunostimulatory potential of LC may be leveraged to develop therapeutic cancer strategies [83].

Migration inhibitory factor (MIF), produced by KC recruits, maintains antigen-presenting cells in the dermis/epidermis. The absence of MIF causes a reduction in the number of skin antigen-presenting cells, which is associated with the accelerated and increased formation of nonmelanoma skin tumors during chemical carcinogenesis [84]. In contrast, MIF(^−/−^) BALB/c mice have a significantly diminished acute inflammatory response to UVB exposure and reduced tumor incidence with significantly less angiogenesis and delayed tumor progression compared to wild-type mice [85]. MIF transgenic mice have a corresponding increased tumor incidence [86]. Because LC are not the only immune cells attracted by MIF [84], additional potential mechanisms for the involvement of MIF in tumorigenesis are possible [87]. Thus, the seemingly controversial result regarding MIF could be partially explained by the use of different tumor models and mouse strains, since BALB/c mice harbor an inactivating mutation of the cyclin-dependent kinase inhibitor p16^INK4a^ [88], which increases the risks of associated cell-cycle alterations in KC. Additionally, MIF has been shown to activate macrophages, T and B cells and to prolong immune cell survival by inhibiting apoptosis.

In a two-stage cutaneous chemical carcinogenesis model, carcinogenesis is first induced by 7,12-dimethylbenz[a]-anthracene (DMBA) and then coupled with repeated applications of a proinflammatory tumor promoter, 12-O-tetradecanoyl-phorbol-13-acetate (TPA) [89]. This skin tumor model was designed to elucidate the role of the interactions between infiltrating immune cells, KC, and local immune populations, such as LC, in the pathophysiology of cutaneous disease and malignancy. DMBA is a prototypic polyaromatic hydrocarbon (PAH), which are chemicals that are prevalent and potent carcinogens present in automobile emissions, tobacco smoke, broiled meat, shellfish, industrial soot, and groundwater [90]. Hence, this model possesses intrinsic and generalizable merits as a model for environmentally-induced cutaneous dysregulation and carcinogenesis. Despite the implicated immunogenic role of LC in immune surveillance and antitumor immunity, mice that are deficient in LC are almost completely resistant to tumor formation in this two-stage carcinogenesis model [91]. Mice with LC deletion are protected from cutaneous chemical carcinogenesis, since LC metabolize PAH into a potentially oncogenic intermediate, which enhance mutagenesis and facilitates epithelial DNA damage and squamous cell carcinoma [92]. In addition, p450 enzyme CYP1B1 produced by LC is required for the maximal induction of DNA-damage within adjacent KC, leading to efficient neoplastic transformation, and tumor progression [93]. Furthermore, studies reveal the capacity of LC to internalize and metabolize PAH, which enhance their role in mutagenicity [94]. Meanwhile, the physical proximity of LC to basal KC raises speculation regarding the possible direct influence of LC on facilitating the survival of genetically transformed KC via the local production of growth factors or anti-apoptotic factors [79]. Overall, these results suggest that LC make multifaceted contributions to cutaneous carcinogenesis by influencing early events in malignant transformation and facilitating cancer cell survival.

Similar to LC in mice, human LC reside in the basal and supra-basal layers of the epidermis, where they form a dense network of first-line defense against invading pathogens. A recent study compared LC frequencies in 41 human non-melanoma tumor lesions and 16 healthy control tissues, revealing significantly decreased LC frequencies in tumor tissue and adjacent epidermis overlying malignant tumor tissue compared to healthy skin; additionally, squamous cell carcinoma (SCC) lesions showed a more dramatic decrease of LC than that of basal cell carcinoma (BCC) lesions [95]. Consistent with this report, a systematic review of available nonmelanoma skin cancer-related studies was performed and quantitatively assessed LC in nonmelanoma skin cancers and identified an overall trend towards slightly higher numbers of LC in BCC than in SCC lesions, although there were discrepancies between individual studies [96]. Human LC pulsed with tumor or viral peptides were superior to CD14^+^ dermal DC in priming high-avidity antigen-specific CD8^+^ CTL that expressed higher levels of granzymes and perforin [97]. Additionally, LC from SCC and peritumoral skin has been shown to induce dramatic T-cell proliferation and IFN-g production, which could not be blocked by tumor supernatant enriched with immune suppressive cytokines. These findings indicate that LC may be superior to DC for use in SCC immunotherapy, providing a new rationale for harnessing LC for the treatment of SCC [98]. The superior immunogenic capacity of LC might be due to the preferential IL-15 production by LC, which is known to enhance naïve CD8^+^ T cell proliferation and stimulate memory T cell development [99]. In clinical trials using an ex vivo monocyte-derived DC vaccine to prevent melanoma, GM-CSF and IL-15 treatment preferentially elicited LC-type DC, which are more efficient in priming melanoma-antigen-specific CD8^+^ T cells in vitro than their monocyte DC counterparts. This priming may result in the generation of powerful CD8^+^ effector cells with an increased ability to target tumor antigens [100,101]. Furthermore, human LC have an advanced capability to prime CD4^+^ naïve T cells relative to other human skin dendritic cells [102,103]. Studies on the role of LC in murine skin tumor models and human skin cancers is summarized in Table 2.

The contradicting roles of LC in carcinogenesis seen in murine PAH-induced skin cancer and inhuman skin cancer patients may be explained in numerous ways. First, the murine and human study models and disease development windows differ substantially; while the murine models of genetic LC deletion focus on the early events in malignant transformation from chemical-induced mutagenesis and UV-induced immune suppression, clinical studies using patient skin cancer samples are fully developed tumors fueled by multiple additional factors not accounted for in the murine models, including, but not limited to, ultraviolet-induced mutagenesis and immunosuppression. Second, the constitutive LC deletion used in the PAH-induced skin cancer mouse model may have an impact on normal skin environment and skin cell homeostasis [32]. Thus, the future use of mouse models that enabled the inducible targeting of LC would be beneficial to the better understanding of the role of LCs in mounting immune responses against malignant transformation or other insults. Third, taking into account the migration capability of LC is critical. LC lost due to severe inflammation can be replenished by bone marrow derived monocytic precursors, whereas the loss of LC due to more mild stimuli can be resolved by the local proliferation of LC. Thus, the composition and the functionality of the LC pool, as well as the LC contribution to immune responses against tumors may vary depending on the experimental models and tumor developmental stages assessed. Finally, although many aspects of LC biology and functionality are comparable between mice and humans [104], profound differences in anatomical and immunological features exist comparing murine and human skin [105]. For instance, unlike in mice, human LC have been reported to be more efficient activators of naïve CD8 T cells than dermal DCs, which might have important implications for antitumor responses [97]. Thus, extrapolating data from murine studies into the understanding of human biology may sometimes be problematic. Diversities in the role of LC in the regulation of human versus murine cutaneous immune responses may exist and may be due to the specificities of immune cell components in the skin, such as T cell types in the epidermis.

## 6. Potential Mechanisms of Langerhans Cells in Priming Treg Responses Versus Effector T Cell Responses

While LC-induced regulatory T cell priming and immune tolerance plays an important role in cutaneous immune homeostasis, LC are also proficient in priming naïve conventional T cells in draining LN. Here, LC provide requisite signals for T cell differentiation into functional effector/memory T cells in response to nocuous antigens during pathogen invasion or inflammation. Furthermore, a unique role of LC in licensing effector function of CD8^+^ T cells in the epidermis has also been demonstrated [106]. Nonetheless, the nature of the signaling and transcriptional programs that regulate and guide the function of LC in tolerance versus immunity induction remain to be elucidated. Ultimately, how LC transcriptional programs during homeostasis are counteracted by pathogen-triggered signals or whether heterogeneous LC populations with distinct tolerogenic or immunogenic functional potential co-exist in the epidermis remain unknown and/or under debate.

The critical involvement of TGF-β in tolerogenic DC induction has been documented in multiple tissues and tumors [107,108,109,110,111]. Additionally, the impact of TGF-β on DC induction of tolerance, such as tumor immune suppression [108], as well as autoimmunity prevention [112], tissue homeostasis [107,111], and inflammation resolution [112], has been demonstrated. Meanwhile, the requirement of TGF-β for TRM-induced Treg differentiation has also been shown in multiple tissues, such as small intestine [33,34] and lung [35,36]. The pivotal role of TGF-β in LC development and epidermal residence has been established, in which TGF-β is absolutely required for LC development [113,114], and the activity of TGF-β is dependent on integrins αvβ6 and αvβ8 that are expressed on KC [115,116]. In addition, TGF-β is required for inflammation-induced LC repopulation, in which alternative TGF-β downstream signaling pathways are involved, which is different from steady-state LC maintenance [22,23]. Given the critical role of TGF-β and specific downstream signaling pathways in the epidermal retention and maintenance of LC, as well as the critical involvement of TGF-β in the induction of tolerogenic function in both DC and TRM in multiple tissues, it is tempting to speculate a potential role for TGF-β in programming the LC tolerogenic state.

Based on recent the genomic and epigenomic analyses of human LC and studies of other types of DC, Polak and Singh hypothesized that human LC tolerogenic and immunogenic states are directed through the counteraction of gene regulatory networks that are coupled to a shared core maturation gene module [117]. The authors proposed that the stability of this functionally specific transcriptional state is controlled by multiple positive and negative regulatory feedback loops. TGF-β activation and signaling facilitated by the KC expression of integrins leads to the retention of immature LC in the epidermis by promoting LC–KC interactions, which prevent LC migration and reinforce the immature LC state with tolerogenic potential [116,118,119]. Nevertheless, the inhibition of TGF-β signaling and/or loss of EpCAM promotes LC migration, maturation, and tolerogenic programming, which is dependent on the activation of β-catenin signaling and the upregulation of interferon regulatory factor 4 (IRF4) [120]. Intriguingly, IRF4 has been shown to be positively regulated post-translationally by the ROCK2 kinase, and this process is reciprocally antagonistic to the E-cadherin molecules, which facilitate attaching LC to the surrounding keratinocytes [118,121]. This raises the possibility that ROCK2 may be similarly regulated by other cell–cell adhesion molecules, such as EpCAM.

The tolerogenic LC transcriptional program consists of a functional gene module that includes the IDO1, LGALS1 (Galectin 1), and interleukin 4-induced 1 (IL-4I1) genes. While LGALS1 and IL-4I1 are directly regulated by IRF4, IDO1 is regulated by IRF4 and ligand-activated AhR [121,122]. Interestingly, AhR expression in human hepatocytes is positively controlled by the β-catenin [123], raising the possibility that it may also occur in LC. A recent study employed Langerin- and CD11c-cre-mediated AhR deletion mice to dissect the role of AhR in the function of LC and dermal DC. This study concluded that deletion of AhR in Langerin-expressing cells diminishes the number and activation of LC, while enhancing Th2 and Tr1 responses upon epicutaneous protein sensitization [124]. These results indicates that AhR may function as a pivotal molecular switch governing the regulatory function of LC [124]. Importantly, kynurenine metabolites, produced by IDO-mediated tryptophan catabolism, could augment AhR activity and sustain IDO expression, creating a positive feedback loop previously proposed to operate in other tolerogenic DC [122,125]. Thus, it has been proposed that a self-reinforcing control circuit involving the loss of EpCAM-mediated LC:KC interactions; the induction of IRF4, ROCK2, and β-catenin signaling; and AhR activation could orchestrate a cell migration-coupled maturation program alongside a tolerance-inducing genomic module in LC [117].

During the infection of the epidermis, TLR and proinflammatory cytokine signaling override TGF-β signaling [126,127]. Polak and Singh have further hypothesized that proinflammatory signaling from pathogen-derived danger signals and activated KC induce an immunogenic LC transcriptional program regulated by IRF1 and NF-κB [117]. Notably, IRF1 and IRF4 have been shown to counter-regulate each other’s functions in a variety of cellular contexts [128,129,130]. Consistent with this hypothesis, TNF signaling results in the induction of IRF1-mediated transcriptional programs in LC, including an upregulated cytotoxic T lymphocyte activities [120]. A recent sc-RNAseq study on human LC from Polak’s group identified the TNF-alpha-enhanced LC immunogenic program, and confirmed that IRF1 is the key transcription factor that regulates LC immunogenicity [131].

**Figure 3:**
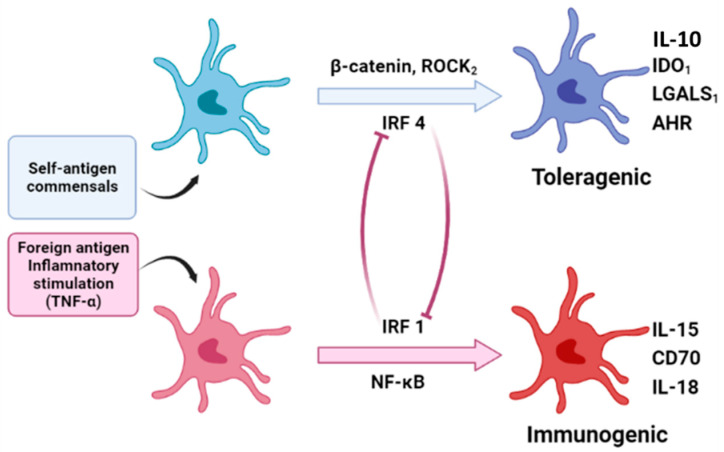
**Schematics for the proposed functional plasticity. Left penal** showed the heterogeneity of LC and the development of tolerogenic and the immunogenic of LC, which could be susceptible to different environment factors. **Middle panel** showed the different signaling pathways mediated the activation and the counter-regulatory role of different types of LC. **Right panel** showed the molecular mechanisms used by LC to induce tolerogenic or immunogenic immune responses.

Meanwhile, heterogeneity within LC population has been shown to be marked by the distinct combinations of transcription factor expression profiles within individual LC, which translates into the differential susceptibility of LC subpopulations toward inflammatory versus tolerogenic signaling and indicates the existence of an in situ LC immunocompetence spectrum [129,131]. In support of this hypothesis, our recent scRNAseq study of purified murine LC at different developmental stages showed the existence of different LC subtypes with diverse transcriptomes that were initiated from the embryonic stage (unpublished results). This result suggests the pre-existence of LC subtypes that possess different functional potential. Taken together, it can be speculated that different types of LC would be susceptible to different environment cues and lead to different immune responses accordingly. Meanwhile, the different types of LC would reciprocally regulate each other and form a counter-regulatory system and be susceptible to different environment cues to form a counter-regulatory system to maintain skin homeostasis under steady state conditions, while maintaining the potential to launch an efficient immunogenic response when encountering pathogen infection and/or malignant transformation (Figure 3). The detailed molecular mechanisms and specific signaling pathways regulating the development, maintenance, and function of different subtypes of LC remain to be further explored.

## 7. Conclusions

In conclusion, LC are a unique constituent of the TRM family. In contrast to other tissue macrophages, LC display a striking combination of macrophage and DC features. In addition to the well-known DC-like immune activation function of LC, recent studies support an immunosuppressive role for LC at steady state and during various inflammatory conditions. Although significant progress has been made in our understanding of the key roles that LC play in cutaneous immunity, the specific immunologic context within the skin that directs LC to either activate or suppress proinflammatory adaptive immune responses remains to be clarified. Understanding how LC integrate different microenvironmental cues to adapt their immunological responses and elucidating the detailed molecular mechanisms and signaling pathways that control LC functional plasticity will be necessary for clarifying the mechanisms of inflammatory skin diseases, enhancing our knowledge of skin tumor immunology, and moving vaccine research forward. Future investigations in this direction will provide exciting new insights into the functional repertoire of LC and contribute to the successful translation of LC biology into clinical application.

## Figures and Tables

**Table 1:** Langerhans cells in UV-mediated immune suppression.

Model Type	Observed LC Function	LC Location	Reference
UVR, Mu-Langerin-DTR	migration and induction of antigen-specific Treg post UVR	LN	[8] Schwarz A et al.
UVR, Mu-Langerin-DTR, Ag-specific CD8T transfer	Dermal Langerin^+^ DC but not LC reduce CD8 T expansion post UVR	LN	[9] Wang L, et al.
UVR, Mu-Langerin-DTR	phagocyte apoptotic KC, anti-inflammation post UVB	Skin	[11] Hatakeyama M et al.
UVR, K14-RANKL^Tg^	RANK (LC)-RANKL (KC) interaction enhance Tregfunction	LN, Skin	[15] Loser K et al. [16] Yoshiki R et al.
UVR, Mu-Langerin-DTR	OX40L (LC)- OX40 (T) interaction induce T reg	LN, Skin	[16] Yoshiki R et al.
CD300a^−/−^ mice	CD300a (LC)-PC (apoptotic KC) interaction inhibit Treg.	Skin	[17] Nakahashi-Oda C et al.
UVR, hu-Langerin DTA	promote p53 mutant KC clonal islands expansion	Skin	[18] Lewis JM et al.
UVR, hu-Langerin DTA	facilitate ILC3 shift enhance mutant KC growth upon UV	Skin	[19] Lewis JM et al.

**Table 2:** Langerhans cells in murine skin tumor models and human skin cancers.

Model Type	Tumor Model	Skin Tumor Development	LC or DC Phenotype	Reference
Mouse MIF KO	Chemical-DMBA-TPA	decreased tumor	decreased skin LC & other immmune cells	[84] 2017
Mouse MIF KO (BALB/C)	UV-induced	Increased tumor	N/A	[85] 2009
Mouse MIF Tg	UV-induced	Decreased tumor	N/A	[86] 2009
Mouse LC deletion	Chemical-DMBA-TPA	Decreased tumor	N/A	[91], 2008
Mouse LC deletion	Chemical-DMBA-TPA	Decreased tumor	LC matablize DMBA, induce Hras mutation	[92], 2012
Mouse LC deletion	Chemical-DMBA-TPA	Decreased tumor	LC originated CYB1P1 required for KC DNA damage	[93], 2015
Human	BCC, SCC	N/A	Decreased LC in BCC/SCC than HC; increased LC in BCC than SCC	[95] 2020
Human	Summary of 30 nonmalanoma skin cancer studies	N/A	Increased LC in BCC than in SCC	[96] 2020
Human	SCC	N/A	SCC LC better inducer of CD4/CD8 T activation than paratumoral LC	[98] 2012
Human	in vitro monocyte differentiation	N/A	IL-15 skew monocytes into LC	[100] 2001
Human	in vitro monocyte differentiation	N/A	LC-like IL15-DC are better inducer of tumor-specific CTL than IL4-DC	[101] 2007
Human	non-tumor skin	N/A	IL-15 by LC induced CD8 T effector function	[99] 2012
Human	in vitro T cell differentiation	N/A	LC are better inducer of CD4 T activation than CD1c+ DDC	[103] 2010
Human	in vitro T cell differentiation	N/A	LC are better than DDC in inducing CD4 T IL21, IL22 production.	[102] 2012

## Data Availability

Not applicable.
